# Transient Gestational Hypertension and Its Subsequent Maternal and Perinatal Outcomes

**DOI:** 10.7759/cureus.116795

**Published:** 2026-09-23

**Authors:** Nicolás García, Paulino Vigil-De Gracia

**Affiliations:** 1 Gynaecology and Obstetrics, Caja de Seguro Social, Panama City, PAN; 2 Obstetrics and Gynaecology, Seguro Social Panamá, Sistema Nacional de Investigadores (SNI) of Secretaria Nacional de Ciencia y Tecnología (SENACYT), Panama City, PAN

**Keywords:** ambulatory blood pressure monitoring, blood pressure, gestational hypertension, preeclampsia, pregnancy, transient gestational hypertension

## Abstract

Objective: To describe maternal and perinatal outcomes among pregnant women with transient gestational hypertension (TGH) defined through prolonged in-hospital surveillance.

Methods: This retrospective cohort included pregnant women hospitalized at ≥20 weeks of gestation at a referral hospital in Panama between July 1, 2023, and December 31, 2024. Women were included if they had an initial systolic blood pressure ≥140 mmHg and/or diastolic blood pressure ≥90 mmHg, with no subsequent elevated reading during seven days of measurements obtained every four hours; they were then discharged and followed until delivery. The primary outcome was the subsequent development of gestational hypertension or preeclampsia.

Results: A total of 20 pregnant women were included; mean age was 31.9 ± 6.3 years, median gestational age at the initial episode was 32.1 weeks (interquartile range (IQR), 27.9-34.2). Five patients (25.0%; 95% confidence interval, 11.2-46.9) subsequently developed a hypertensive disorder: three developed gestational hypertension and two developed preeclampsia, including one with severe features due to a hypertensive crisis. Among those who progressed, the median time to the new diagnosis was 30 days (IQR, 27-35). Delivery occurred at a median of 38 weeks; there was one preterm delivery, one neonatal intensive care unit admission, and no maternal or perinatal deaths. In exploratory analyses, progression was associated with a higher cesarean delivery rate and lower neonatal birth weight.

Conclusions: Even after seven days of in-hospital normotension, one in four pregnant women progressed to TGH. TGH should not be considered benign and requires subsequent surveillance, ideally with out-of-hospital blood pressure measurements.

## Introduction

Hypertensive disorders of pregnancy (HDP) constitute a dynamic clinical continuum. An initial elevated blood pressure reading after 20 weeks may mark the onset of persistent gestational hypertension or preeclampsia, but it may also normalize during the initial observation period. Accurate classification of this episode is important because imprecise labeling can lead both to unnecessary interventions and to false reassurance.

The International Society for the Study of Hypertension in Pregnancy (ISSHP) distinguishes between two entities that are frequently confused: white-coat hypertension is defined as clinic blood pressure ≥140/90 mmHg before 20 weeks, with normal values on home or ambulatory monitoring; in contrast, transient gestational hypertension occurs at or after 20 weeks and resolves with repeated measurements, usually over several hours. The latter has been associated with a risk of nearly 40% of subsequently developing established gestational hypertension or preeclampsia and therefore should not be considered a benign phenomenon [1,2].

Specific evidence is limited. Hawkins and colleagues published the reference cohort on transient gestational hypertension and observed subsequent progression to gestational hypertension in 20% and to preeclampsia in 19% of cases [2]. In that study, transience was established after an initial 4-6-hour assessment. No maximum interval has been established for ruling out or confirming this hypertensive disorder of pregnancy [1].

Terminologic confusion persists even in recent research. Espeche and colleagues studied high-risk women between 20 and 34 weeks and referred to clinic hypertension with nonsustained ambulatory blood pressure as white-coat hypertension [3]. According to the ISSHP temporal classification, patients with a de novo elevation after 20 weeks and normal ambulatory blood pressure align more closely with the concept of transient gestational hypertension; in that study [3], those with nocturnal hypertension did not have completely normal blood pressure outside the office and therefore do not represent classic white-coat hypertension. Nevertheless, that study provides an important message: nocturnal blood pressure may identify a high-risk subgroup that is not recognized by isolated clinic measurements [3].

The objective of this study was to describe maternal and perinatal outcomes among pregnant women at 20 weeks of gestation or later who presented with an initial blood pressure elevation, remained normotensive during seven days of in-hospital surveillance, and were subsequently discharged. The primary outcome was the subsequent development of gestational hypertension or preeclampsia.

## Materials and methods

Study design and setting

An observational retrospective cohort study was conducted at the Hospital Complex of the Caja de Seguro Social of Panama, a national referral hospital for high-risk pregnancies. The inclusion period extended from July 1, 2023, through December 31, 2024.

Ethical considerations

The study was approved by the Ethics Committee of the Hospital Complex of the Caja de Seguro Social of Panama, approval no. CBI-CHDrAAM-09-2026. Because this was a retrospective medical-record review, the information was analyzed anonymously. A waiver of informed consent was approved by the committee.

Study population and selection criteria

Hospitalized women at ≥20 weeks of gestation were included after a first documented blood pressure elevation, defined as systolic blood pressure ≥140 mmHg and/or diastolic blood pressure ≥90 mmHg. Patients were excluded if they had a second elevated reading during the initial admission, a confirmed diagnosis of gestational hypertension or preeclampsia during that hospitalization, a known history of chronic hypertension according to the medical record, no follow-up through delivery, or insufficient data to establish the outcome.

The database contained 20 eligible records; all were within the study period and had follow-up through delivery.

Operational definition of transient gestational hypertension

For this study, transient gestational hypertension was defined as an initial systolic blood pressure ≥140 mmHg and/or diastolic blood pressure ≥90 mmHg occurring after 20 weeks, followed by seven complete days of in-hospital surveillance with measurements every four hours and no subsequent hypertensive reading. Some physicians use the term white-coat hypertension for this same condition. After persistent hypertension and preeclampsia had been ruled out during that admission, the patients were discharged and continued obstetric follow-up until delivery. This operational definition requires a much longer period than the resolution over several hours described by the ISSHP and the initial 4-6-hour assessment used in the Hawkins cohort; it has not been validated as a universal diagnostic criterion [1,2].

Variables and outcomes

The following variables were extracted: maternal age, gravidity, primigravida status, abortion in previous pregnancies, gestational age at the index episode, pattern of the initial blood pressure elevation, 24-hour proteinuria, antenatal corticosteroid administration, subsequent hypertensive diagnosis, interval to admission or diagnosis, gestational age and mode of delivery, indication for delivery, neonatal birth weight, Apgar score, admission to the neonatal intensive care unit (NICU), mechanical ventilation, sepsis, and hospital length of stay.

The primary outcome was the subsequent development of any HDP, defined as gestational hypertension or preeclampsia documented in the medical record. Secondary outcomes were preeclampsia with severe features, preterm delivery at <37 weeks, cesarean delivery, low birth weight < 2500 g, NICU admission, and maternal or perinatal death.

Statistical analysis

Statistical analyses were performed using Epi Info™ version 7.2.7 (Centers for Disease Control and Prevention, Atlanta, GA). Continuous variables were summarized as mean and standard deviation (SD) or median and interquartile range (IQR), as appropriate; categorical variables were expressed as frequency and percentage. The 95% confidence interval (CI) for the proportion with the primary outcome was calculated using the Wilson method. Comparisons between patients with and without progression were exploratory and used the Fisher exact test for categorical variables and the Mann-Whitney test for continuous variables. Multivariable modeling was not performed because of the small sample size. A p-value < 0.05 was considered statistically significant, without adjustment for multiple comparisons; therefore, these results were interpreted as hypothesis-generating.

## Results

A total of 20 records were identified, and all met the eligibility criteria. The mean age was 31.9 ± 6.3 years; nine women (45.0%) were primigravidas, and six (30.0%) had a history of abortion in previous pregnancies. Median gestational age at the initial episode was 32.1 weeks (IQR, 27.9-34.2). The initial elevation was isolated systolic in five patients (25.0%), isolated diastolic in 11 (55.0%), and combined in four (20.0%). Median 24-hour proteinuria was 190 mg (IQR, 132-232); three patients had values ≥ 300 mg/24 hours without persistent hypertension during the initial observation period.

During follow-up, five patients developed an HDP (25.0%; 95% CI, 11.2-46.9): three developed gestational hypertension (15.0%) and two developed preeclampsia (10.0%). One patient with preeclampsia had a hypertensive crisis and was classified as having preeclampsia with severe features (Table 1).

**Table 1:** Study findings (n = 20). HDP, hypertensive disorders of pregnancy; 95% CI, 95% confidence interval; NICU, neonatal intensive care unit.

Outcome	Result
Any subsequent HDP, n (%)	5 (25.0); 95% CI, 11.2-46.9
Gestational hypertension, n (%)	3 (15.0)
Preeclampsia without severe features, n (%)	1 (5.0)
Preeclampsia with severe features/hypertensive crisis, n (%)	1 (5.0)
No subsequent HDP, n (%)	15 (75.0); 95% CI, 53.1-88.8
Gestational age at delivery, weeks, median (IQR)	38 (37–39)
Cesarean delivery, n (%)	6 (30.0)
Preterm delivery <37 weeks, n (%)	1 (5.0)
Neonatal birth weight, g, mean ± SD	3120 ± 468
Low birth weight <2500 g, n (%)	2 (10.0)
5-minute Apgar score, median (IQR)	9 (9-9)
NICU admission, n (%)	1 (5.0)
Mechanical ventilation, n (%)	1 (5.0)
Neonatal sepsis, n (%)	1 (5.0)

Among those who progressed, the median time to the new diagnosis was 30 days (IQR, 27-35). Patients who progressed to an HDP had a lower neonatal birth weight than those without progression (median, 2890 vs 3240 g; p=0.025) and a higher cesarean delivery rate (80.0% vs 13.3%; p=0.014) (Table 2). No differences were detected in maternal age, gestational age at the initial episode, primigravidity, abortion in previous pregnancies, initial proteinuria, or time to rehospitalization. These comparisons were exploratory, had limited statistical power, and were not corrected for multiplicity.

**Table 2:** Findings according to progression to a hypertensive disorder of pregnancy (n = 20). Data are presented as median (IQR) or n (%). Continuous variables were compared using the Mann-Whitney U test and categorical variables using Fisher’s exact test, as appropriate. For Fisher’s exact test, no χ² statistic is reported. A two-sided p-value <0.05 was considered statistically significant. IQR, interquartile range; NICU, neonatal intensive care unit.

Variable	Progression (n=5)	No progression (n=15)	T statistic	p
Maternal age, years, median (IQR)	30.0 (29.0-34.0)	33.0 (29.0-37.0)	U = 30.5	0.569
Gestational age at initial episode, weeks, median (IQR)	32.1 (28.0-34.0)	32.1 (27.9-34.8)	U = 32.5	0.694
Proteinuria, mg/24 h, median (IQR)	214 (200-226)	180 (126-225)	U = 26	0.336
Cesarean delivery, n (%)	4 (80.0)	2 (13.3)	- (Fisher exact)	0.014
Gestational age at delivery, weeks, median (IQR)	37.0 (37.0–38.0)	38.0 (37.0–39.0)	U = 21	0.144
Neonatal birth weight, g, median (IQR)	2890 (2820–2955)	3240 (3065–3458)	U = 12	0.025
Preterm delivery, n (%)	1 (20.0)	0	- (Fisher exact)	0.25
NICU admission, n (%)	1 (20.0)	0	- (Fisher exact)	0.25

## Discussion

The main finding of this cohort was that 25% of pregnant women subsequently developed gestational hypertension or preeclampsia, even after remaining for seven days with measurements every four hours and no second hypertensive reading. Prolonged normalization during hospitalization reduced the likelihood of immediate sustained hypertension but did not eliminate the risk of subsequent disease. The development of preeclampsia with severe features due to a hypertensive crisis reinforces that this phenotype should not be considered benign.

The observed proportion was lower than the combined risk of approximately 39-40% reported by Hawkins and incorporated into the ISSHP recommendations [1,2]. Hawkins reported progression to gestational hypertension in 20% and to preeclampsia in 19% of women with transient gestational hypertension [2]. A plausible explanation is the difference in the confirmation period: the initial assessment in that study lasted 4-6 hours, whereas our protocol required seven complete days of in-hospital normotension. Therefore, the present study probably selected a subgroup at lower immediate risk. Nevertheless, progression in one of every four patients shows that even a much stricter operational definition does not identify a risk-free population.

Nomenclature is essential. Classic white-coat hypertension implies clinic hypertension before 20 weeks with normal blood pressure outside the healthcare setting [1,4]. De novo hypertension after 20 weeks that resolves with repeated measurements corresponds to transient gestational hypertension. Therefore, labeling patients identified in the second half of pregnancy as having “white-coat” hypertension combines phenomena with different temporal and possibly pathophysiologic bases and makes comparisons across studies difficult [3-7]. A study by Espeche and colleagues illustrates this difficulty [3]. The authors evaluated 991 high-risk pregnant women between 20 and 34 weeks; among 79 with clinic hypertension, they classified 40 as having sustained hypertension, 28 as having white-coat hypertension without nocturnal hypertension, and 11 as having white-coat hypertension with nocturnal hypertension. Nocturnal hypertension was independently associated with preeclampsia (adjusted odds ratio, 11.95) and conferred a risk similar to that of sustained hypertension [3]. Under a strict reading of the ISSHP classification, the group with normal ambulatory blood pressure after 20 weeks overlaps more closely with transient gestational hypertension or isolated office gestational hypertension than with classic white-coat hypertension. In turn, the group with nocturnal hypertension does not meet the requirement for normal blood pressure outside the office and represents an occult ambulatory hypertensive phenotype. This reinterpretation does not diminish the relevance of the study; rather, it shows that ambulatory monitoring can reclassify risk that is not detected by clinic measurements.

Our protocol incorporated measurements every four hours throughout the hospitalization and therefore yielded a large number of observations. However, in-hospital surveillance is not equivalent to home blood pressure monitoring or 24-hour ambulatory blood pressure monitoring (ABPM). The hospital environment may alter sleep, activity, and autonomic responses; moreover, it cannot establish that blood pressure is normal in everyday life. ABPM provides daytime, nighttime, and circadian information, whereas home blood pressure monitoring allows repeated surveillance after discharge. The ISSHP recommends additional follow-up, ideally with home blood pressure monitoring, for transient gestational hypertension [1,8-10]. Twenty-four-hour ABPM better distinguishes sustained hypertension from isolated clinic elevation and has demonstrated prognostic value in the third trimester [11,12]. In addition, expert consensus favors measurements 2-3 days per week after 20 weeks [4,13,14].

In the present study, patients who progressed had more cesarean deliveries and lower neonatal birth weight. These associations should be interpreted cautiously: the small sample size, lack of adjustment, and possibility of reverse causation preclude considering them predictors. They likely reflect the indication for delivery and morbidity resulting from the subsequent hypertensive disorder. The only patient with preeclampsia with severe features accounted for the delivery at 33 weeks, the lowest neonatal birth weight, and the NICU admission.

Strengths include complete follow-up through delivery, management under a uniform seven-day protocol with serial measurements, and the description of real-world practice at a Latin American referral hospital, a setting that is underrepresented in the literature. Limitations include the retrospective design, small sample size, absence of a normotensive or sustained-hypertension comparison group, lack of ambulatory or home monitoring, inability to validate the measurement technique and device in all medical records, and absence of potentially relevant variables such as body mass index, history of preeclampsia, diabetes, kidney disease, aspirin use, and angiogenic markers.

Clinically, a patient whose blood pressure normalizes after 20 weeks may be discharged once preeclampsia and persistent hypertensive disease have been ruled out, but discharge should not be equated with diagnostic closure. The condition should be documented as transient gestational hypertension, close maternal and fetal surveillance should be established, and home or ambulatory blood pressure monitoring should be incorporated when available. Prospective studies should compare an initial 4-6-hour observation period with prolonged surveillance and determine whether seven days of hospitalization provide sufficient prognostic benefit to justify their cost and resource utilization.

## Conclusions

Transient gestational hypertension is not a benign finding, even when initial normalization persists for a prolonged period and the classification as white-coat hypertension after 20 weeks is conceptually imprecise; the term transient gestational hypertension better describes this phenotype. Prospective cohorts with home or ambulatory monitoring, comparison groups, and uniform criteria are needed to establish the most efficient diagnostic and follow-up strategy.

## References

[1] Magee LA, Brown MA, Hall DR (2022). The 2021 International Society for the Study of Hypertension in Pregnancy classification, diagnosis and management recommendations for international practice. Pregnancy Hypertens.

[2] Lee-Ann Hawkins T, Brown MA, Mangos GJ, Davis GK (2012). Transient gestational hypertension: Not always a benign event. Pregnancy Hypertens.

[3] Espeche W, Minetto J, Cerri G (2026). Does nocturnal hypertension reclassify maternal-fetal risk in women with white-coat hypertension? A cohort study in the second half of pregnancy. Hypertens Res.

[4] Johnson S, Gordijn S, Damhuis S (2022). Diagnosis and monitoring of white coat hypertension in pregnancy: an ISSHP consensus Delphi procedure. Hypertension.

[5] Johnson S, Liu B, Kalafat E, Thilaganathan B, Khalil A (2020). Maternal and perinatal outcomes of white coat hypertension during pregnancy: a systematic review and meta-analysis. Hypertension.

[6] Roberts JM, Countouris M (2020). White coat hypertension in pregnancy: the challenge of combining inconsistent data. Hypertension.

[7] Vigil-De Gracia P, García N, Araúz AA (2026). Initial recruitment, diagnosis, conservative management, maternal and perinatal outcomes of hypertensive disorders of pregnancy. AJOG Glob Rep.

[8] Salazar MR, Espeche WG, Balbín E (2019). Office blood pressure values and the necessity of out-of-office measurements in high-risk pregnancies. J Hypertens.

[9] Salazar MR, Espeche WG, Leiva Sisnieguez CE (2019). Nocturnal hypertension in high-risk mid-pregnancies predict the development of preeclampsia/eclampsia. J Hypertens.

[10] Espeche WG, Salazar MR (2023). Ambulatory blood pressure monitoring for diagnosis and management of hypertension in pregnant women. Diagnostics (Basel).

[11] Bellomo G, Narducci PL, Rondoni F, Pastorelli G, Stangoni G, Angeli G, Verdecchia P (1999). Prognostic value of 24-hour blood pressure in pregnancy. JAMA.

[12] Shahbazian N, Shahbazian H, Mohammadjafari R, Mousavi M (2013). Ambulatory monitoring of blood pressure and pregnancy outcome in pregnant women with white coat hypertension in the third trimester of pregnancy. J Nephropharmacol.

[13] Brown MA, Mangos G, Davis G, Homer C (2005). The natural history of white coat hypertension during pregnancy. BJOG.

[14] Kalafat E, Mir I, Perry H, Thilaganathan B, Khalil A (2018). Is home blood-pressure monitoring in hypertensive disorders of pregnancy consistent with clinic recordings?. Ultrasound Obstet Gynecol.

